# Predictive value of neutrophil to lymphocyte ratio and platelet to lymphocyte ratio in advanced hepatocellular carcinoma patients treated with anti–PD‐1 therapy

**DOI:** 10.1002/cam4.3135

**Published:** 2020-05-18

**Authors:** Sirish Dharmapuri, Umut Özbek, Jung‐Yi Lin, Max Sung, Myron Schwartz, Andrea D. Branch, Celina Ang

**Affiliations:** ^1^ Department of Medicine Division of Medical Oncology Tisch Cancer Institute Icahn School of Medicine at Mount Sinai New York NY USA; ^2^ Department of Population Health Science and Policy Tisch Cancer Institute Icahn School of Medicine at Mount Sinai New York NY USA; ^3^ Department of Surgery Recanati/Miller Transplant Institute Icahn School of Medicine at Mount Sinai New York NY USA; ^4^ Department of Medicine Division of Liver Disease Icahn School of Medicine at Mount Sinai New York NY USA

**Keywords:** biomarkers, hepatocellular carcinoma, immunotherapy, neutrophil‐lymphocyte ratio, NLR, platelet‐lymphocyte ratio, PLR

## Abstract

**Background:**

Currently, there are no recognized or validated biomarkers to identify hepatocellular carcinoma patients (HCC) likely to benefit from anti–PD‐1 therapy. We evaluated the relationship between neutrophil‐lymphocyte ratio (NLR) and platelet‐lymphocyte ratio (PLR) and survival outcomes, pretreatment and after three doses (posttreatment) of nivolumab in HCC patients.

**Methods:**

Medical records of HCC patients treated with nivolumab between June 2016 and July 2018 were reviewed. Kaplan‐Meier analysis and the log‐rank test were used to calculate and compare overall survival between NLR < 5 Vs ≥ 5 and among PLR tertiles.

**Results:**

A total of 103 patients were identified. Median age was 66 (29‐89) years. Median treatment duration was 26 (2‐149) weeks. Sixty‐four (62%) patients had Child‐Pugh class A (CP‐A) liver function. Barcelona Clinic Liver Cancer stage was B in 20 (19%) and C in 83 (81%) patients. CP‐A patients who achieved a partial or complete response had significantly lower posttreatment NLR and PLR (*P* < .001 for both) compared to patients who had stable disease or progression of disease. No relationship was observed between response and pretreatment NLR and PLR. NLR < 5 was associated with improved OS compared to NLR ≥ 5 both pretreatment (23 Vs10 months, *P* = .004) and posttreatment (35 Vs 9 months, *P* < .0001). Survival also differed significantly among PLR tertiles both pre‐ (*P* = .05) and posttreatment (*P* = .013). In a multivariable model, posttreatment NLR (HR = 1.10, *P* < .001) and PLR (HR = 1.002, *P* < .001) were strongly associated with survival. In a composite model of posttreatment NLR and PLR, a combination of high NLR and PLR was associated with an eightfold increased risk of death (HR = 8.3, *P* < .001).

**Conclusions:**

This study suggests a strong predictive role of these inflammatory cell ratios in the posttreatment setting in HCC patients treated with anti anti–PD‐1 therapy and should be evaluated in a larger cohort.

## Graphical abstract

In this study evaluating 103 patients with advanced hepatocellular carcinoma, lower neutrophil‐lymphocyte ratio (NLR) and platelet‐lymphocyte ratio (PLR) were both strongly predictive of improved survival after three cycles of nivolumab. Baseline NLR was not associated with survival. An elevated NLR and PLR in the posttreatment setting may be an early sign of treatment failure and may warrant consideration for early change in therapy.

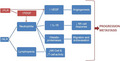

## 1 INTRODUCTION

An evolving understanding of the molecular pathogenesis of hepatocellular carcinoma (HCC) and its complex interactions with the tumor microenvironment has led to the introduction of immunotherapy in the HCC treatment landscape. Nivolumab and pembrolizumab are IgG4 monoclonal antibodies to PD‐1 that are FDA approved for second line therapy after sorafenib. These drugs have not only demonstrated durable responses and prolonged survival in a subset of patients, but also have a better safety profile over sorafenib.^1^, ^2^, ^3^, ^4^ The downside, however, is the unsustainable cost burden associated with anti–PD‐1 therapy, as well as the life‐threatening array of immune‐mediated toxicities observed in about 2%‐4% of patients,^5^ validating the need for development of predictive biomarkers to identify patients that are likely to benefit from anti–PD‐1 therapy.

Unlike neoplasms of the lung, urothelial tract, and stomach where PD‐L1 expression is an FDA‐approved companion biomarker of responsiveness to anti–PD‐1 antibodies, there are no recognized or validated biomarkers in HCC. Responses to anti–PD‐1 therapy have been observed irrespective of baseline PD‐L1 expression ^1^, ^3^, ^6^, ^7^ suggesting its limited sensitivity as a predictive biomarker in HCC. Mismatch repair protein (MMR) deficiency or microsatellite instability (MSI) is an FDA‐approved biomarker for anti–PD‐1 therapy in several advanced solid tumors, but its utility in HCC is limited by the very low frequency of MSI‐High tumors.^7^, ^8^ Tumor mutation burden (TMB) appears to have limited utility as well, given tumor heterogeneity and lack of standardized testing.^7^

Several studies have evaluated the role of inflammatory cell ratios such as neutrophil‐lymphocyte ratio (NLR) and platelet‐lymphocyte ratio (PLR) as predictive biomarkers in patients with other solid tumors treated with anti–PD‐1 therapy (Table 1).^1^, ^9^, ^10^, ^11^, ^12^, ^13^, ^14^ Higher NLR and PLR at baseline are associated with treatment failure and increased risk of death.^11^, ^12^, ^13^, ^15^ Conversely, a lower NLR after several cycles of anti–PD‐1 therapy, as well as a dynamic decrease in NLR with treatment are associated with improved survival.^11^, ^15^ NLR has also been shown to have a strong prognostic potential in a variety of solid tumors across studies.^16^ Here, we studied the predictive value of NLR and PLR on survival outcomes in advanced hepatocellular carcinoma (aHCC) patients treated with nivolumab at the Mount Sinai Hospital.

**Table 1:** Select studies of prognostic and predictive value of NLR and PLR in solid tumors

Study	Tumor	NLR cutoff	NLR Predictive	NLR Prognostic	PLR cutoff		PLR predictive	PLR prognostic
Zaragoza, J et al^1^	Melanoma	≥4		✓				
Cassidy, et al^9^	Melanoma	≥5	✓					
Rosner, S., et al^10^	Melanoma	>4.73	✓					
Ferrucci, et al^11^	Melanoma	≥3	✓	✓				
Bartlett, et al^12^	Melanoma	≥5		✓				
Diem, S, et al^13^	NSCLC	>5		✓	>262			✓
Bilen, MA, et al^14^	Mixed	Log(NLR) = 1.08	✓		Log(PLR) = 5.5		✓	
Howard, R, et al^16^	Mixed	>3.22		✓				
Alagappan, et al^17^	Pancreas	>5		✓				
Cedrés, S, et al^18^	NSCLC	≥5		✓				
Giordano, G et al^19^	Pancreas	≥5	✓	✓				
Pinato, DJ et al^20^	NSCLC	>5	✓		≥300			Negative
Templeton et al^22^	CRPC	>5		✓				
Sacdalan, D B^29^	Mixed Meta‐analysis	variable	✓	✓				
Miyamoto, et al^30^	Gastric	>3.50	✓					

Abbreviations: CRPC, Castrate Resistant Prostate Cancer; NSCLC‐ Non‐Small Cell Lung Cancer.

## 2 METHODS

### 2.1 Patients

Institutional review board approval was obtained to review the medical records of consecutive patients with aHCC treated with nivolumab between June 2016 and July 2018 at the Mount Sinai Hospital. Demographic and clinical data including Barcelona Clinic Liver Cancer (BCLC) stage, Child‐Pugh Score, prior systemic and locoregional therapies, nivolumab treatment duration, best response, follow‐up, and vital status were collected. Tumor assessment was performed at baseline, and thereafter at the discretion of the treating physician at regular intervals. Radiographic responses were classified according to response evaluation criteria in solid tumors (RECIST 1.1).

### 2.2 NLR and PLR

Neutrophil‐lymphocyte ratio was calculated as the ratio of absolute neutrophil count to absolute lymphocyte count (ALC), and PLR was calculated as the ratio of platelet count to ALC. Neutrophil‐lymphocyte ratio and PLR were documented at baseline (pretreatment) prior to the first infusion of nivolumab and after completion of cycle three (posttreatment). A cutoff of five was used for NLR groups based on literature.^17^, ^18^, ^19^, ^20^, ^21^, ^22^ Cutoffs for three‐level groups of PLR were determined at tertiles while cutoff for binary groups of PLR was identified using K‐means clustering^23^; the cutoff was determined to be 500 (<500 n = 88 & ≥500 n = 10). Tertiles were used when describing survival based on prior literature,^13^, ^14^ but this was limited to binary groups in the NLR/PLR model described below to optimize the number of groups created and to power each group adequately.

### 2.3 Statistical analysis

Descriptive statistics were calculated to summarize baseline status, including demographics, disease characteristics, and treatment characteristics. Kruskal‐Wallis, chi‐squared, and Fisher's exact tests were conducted to identify associations between PLR, NLR, and baseline characteristics and tumor response. The objective response rate (ORR) was defined as the proportion of patients with best responses as complete response (CR) or partial response (PR) and the disease control rate (DCR) was defined as proportion of patients with CR, PR, or stable disease (SD). Durations of response were calculated as time from the date of best response to the date of progression or last follow‐up date for patients with objective response.

Overall survival (OS) was calculated from the first cycle of nivolumab to death and progression‐free survival (PFS) was determined from the first cycle of nivolumab to disease progression documented by imaging, or death. The Kaplan‐Meier (KM) method was used to estimate survival of groups, and log‐rank test was employed to compare the KM curves between different groups. Univariable and multivariable Cox proportional hazard (Cox PH) models with NLR and PLR treated as continuous covariates were fitted to investigate associations of NLR and PLR with OS. We also fitted a multivariable Cox PH model controlling for Child‐pugh score and BCLC stage with a composite variable of NLR and PLR to explore the association between interaction of NLR and PLR and OS. To create the composite variable, we first converted NLR and PLR to discrete variables based on the cutoffs described above. The composite variable was then categorized as low NLR/low PLR, high NLR/low PLR, and high PLR (due to sample size and modeling issues, low NLR/high PLR and high NLR/high PLR groups were combined.). Results from Cox PH models were presented as hazard ratios (HR) with 95% confidence intervals (CIs). All analyses were done using R 3.6.1 (Vienna, Austria).^24^

## 3 RESULTS

### 3.1 Patient and disease characteristics

In total, 103 patients were included. Demographics and disease characteristics are summarized in Table 2. The median age was 66 years (range 29‐89) and with a predominantly male population (84%). Racial distribution was notably proportionate between Asian (23%), black (24%) and white (29%) populations. Sixty‐four (62%) patients had Child‐Pugh class A liver function. The most common underlying chronic liver disease was hepatitis C virus (HCV) infection in 50 (49%) patients, followed by hepatitis B virus (HBV) infection in 33(32%). Barcelona Clinic Liver Cancer (BCLC) stage was C in 83 (81%) patients and B in 20 (19%).

**Table 2:** Baseline patient characteristics

	Stable disease	Partial/Complete response	Progression of disease	All subjects	*P*‐value
Age, median (Range), y	67 (29‐83)	71 (30‐89)	64 (30‐85)	66 (29‐89)	**.040**
Number (%)	40 (38.4)	21 (20.1)	38 (37.5)	103^a^(100)	
Gender					.826
Male	33 (82.5)	17 (81.0)	33 (86.8)	86 (83.5)	
Female	7 (17.5)	4 (19.0)	5 (13.1)	17 (16.5)	
Race					**.021**
Asian	10 (25.0)	10 (47.6)	3 (7.8)	24 (23.3)	
Black	8 (20.0)	2 (9.5)	12 (31.5)	25 (24.2)	
White	11 (27.5)	8 (38.0)	11 (28.9)	30 (29.1)	
Other/Mixed	7 (17.5)	1 (4.7)	9 (23.6)	17 (16.5)	
Unknown	4 (10.0)	0 (0)	3 (7.8)	7 (6.7)	
Ethnicity					
Hispanic	5 (12.5)	1 (4.8)	7 (18.4)	13 (12.6)	.443
Non‐Hispanic	34 (85.0)	20 (95.2)	31 (81.5)	89 (86.4)	
Unknown	1 (2.5)	0 (0)	0 (0)	1 (0.9)	
Baseline AFP	22.0 (2.0, 20 000.0)	55.0 (2.0, 65 860.0)	525.0 (2.3, 880 018.0)	113.0 (2.0, 880 018.0)	**.009**
Child‐Pugh Class					.990
A	26 (65.0)	13 (61.9)	24 (63.1)	64(62.1)	
B	11 (27.5)	7 (33.3)	12 (31.5)	32(31.1)	
Unknown	3 (7.5)	1 (4.7)	2 (5.2)	7(6.8)	
Risk factors					
Hepatitis C^b^	23 (57.5)	11 (52.4)	15 (39.5)	50 (48.5)	.284
Hepatitis B^b^	11 (27.5)	9 (42.8)	12 (31.6)	33 (32.0)	.489
NASH	6 (15.0)	1 (4.7)	3 (7.9)	10 (9.7)	.512
Alcohol	2 (5.0)	1 (4.7)	4 (10.5)	8 (7.8)	.601
None	2 (5.0)	1 (4.7)	4 (10.5)	8 (7.8)	.601
Other	2 (5.0)	0 (0)	8 (21.1)	10 (9.7)	**.018**
No. of Risk Factors, median (Range)	1 (0‐2)	1 (0‐2)	1 (0‐2)	1 (0‐2)	.873
Cirrhosis					.853
Yes	33 (82.5)	16 (76.2)	30 (78.9)	82 (79.6)	
No	7 (17.5)	5 (23.8)	8 (21.0)	21 (20.4)	
BCLC Stage					**.009**
B	12 (30.0)	6 (28.6)	2 (5.2)	20 (19.4)	
C	28 (70.0)	15 (71.4)	36 (94.7)	83 (80.6)	

Abbreviations: AFP, Alpha Fetoprotein; BCLC, Barcelona Clinical Liver Cancer Staging; NASH, Nonalcoholic Steatohepatitis.

Statistically significant values are in bold.

^a^ Four patients were not evaluated for Response.

^b^ Co‐Infection: SD ‐ four patients, PR ‐ two patients, POD ‐ one patient

An NLR of < 5 was noted in 64 (62%) patients in the pretreatment setting and 59 (60%) patients in the posttreatment setting. An even distribution of patients was noted among the PLR tertiles in both pre‐ (<119(36%); ≥ 119 & <224(31%); ≥224(33%)) and posttreatment (<126(35%); ≥126 & <229(31%); ≥229(33%)) settings. Demographics and disease characteristics, including age, race, risk factors, the presence of cirrhosis, and BCLC stage, did not differ significantly among NLR groups or PLR tertiles.

### 3.2 Treatment characteristics

Sixty‐six (64%) patients received nivolumab in first line. Among the 37 (36%) treated with nivolumab in subsequent lines, 28 (76%) had previously progressed on sorafenib. The median duration of treatment was 26 (range 2‐149) weeks. Locoregional therapies including radioembolization and chemoembolization were given concurrently with nivolumab in 32 (31%) patients, including all 10 patients with CR.

### 3.3 Response to nivolumab

The ORR was 20% and DCR was 58%. Median duration of response was unreachable as there were only two patients who progressed after an initial response. The crude median duration of response was 6.7 (range 3.5‐10) months. Progression of disease (PD) occurred in 38% of patients who received nivolumab.

**Figure 1:**
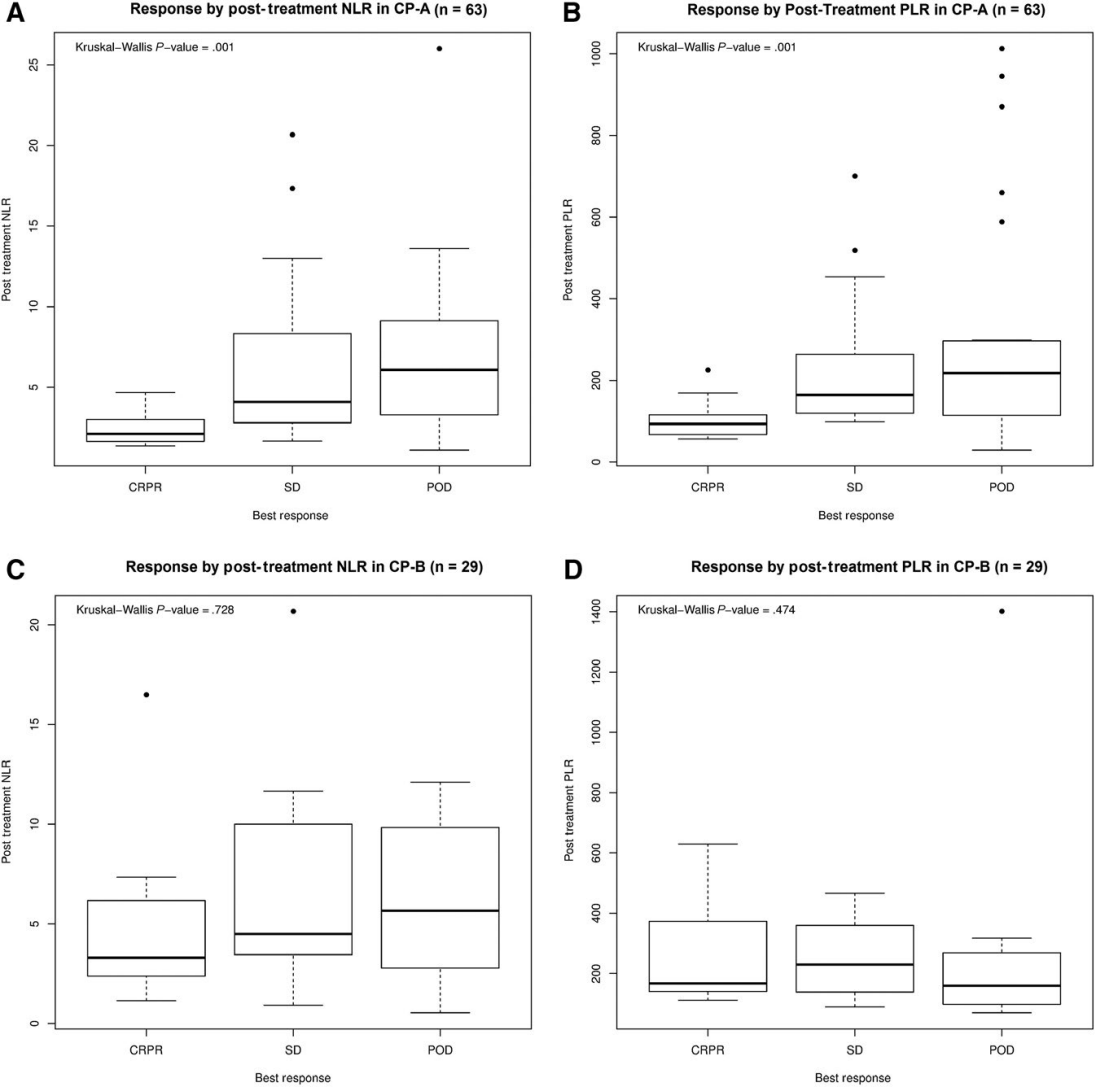
Response by child‐pugh class

Significant differences in baseline characteristics including age, race, baseline AFP, and BCLC stage were observed between response groups (Table 2). ORR did not differ between patients with Child‐Pugh A vs B liver disease (21% vs 23%, *P* = .979). The relationship between posttreatment NLR and PLR and response to nivolumab was significant in patients with Child‐Pugh A disease (Figure 1; *P* < .001 for both NLR and PLR) but not in patients with Child‐Pugh B disease (*P* = .474 for PLR and *P* = .728 for NLR). Child‐Pugh A patients who achieved a partial or complete radiographic response had significantly lower posttreatment NLR and PLR compared to patients who had SD or PD while there were no significant differences in posttreatment NLR and PLR between patients with SD and with PD. No relationship was observed between response and pretreatment NLR and PLR.

### 3.4 Survival

**Figure 2:**
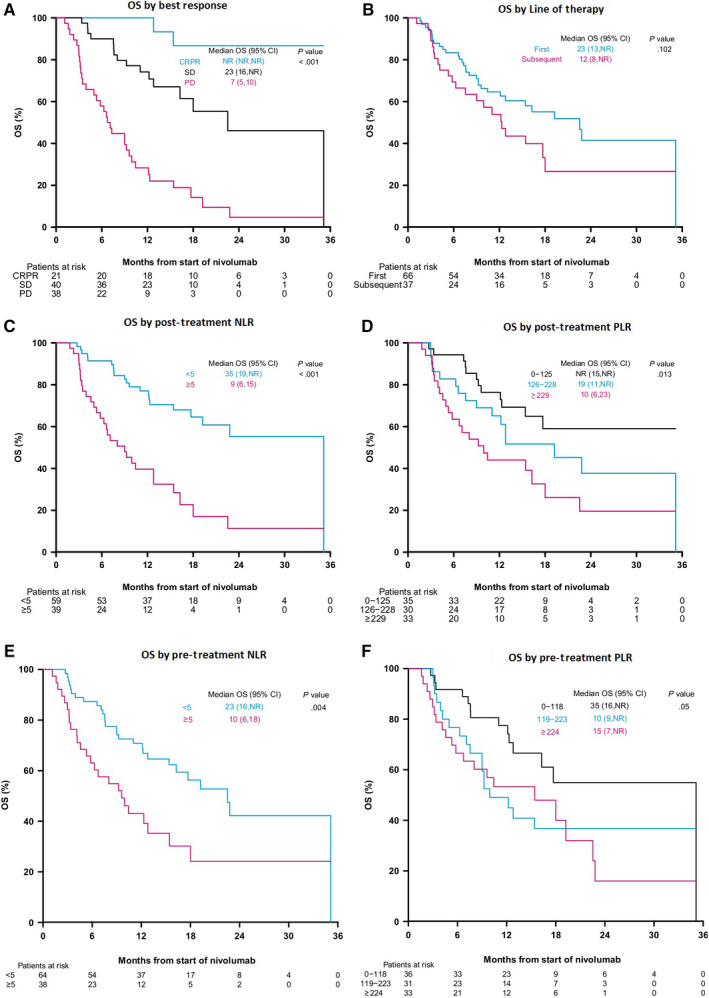
Overall survival

The median follow‐up was 17 months (95% CI 15.7, 20.7). The median OS for the study population was 16 months (95% Cl: 12 ‐ Not reached). Median OS was longer in Child‐Pugh A vs B patients (23 vs 9 months). Overall survival differed significantly by best response (*P* < .001), but not by line of therapy (Figure 2).

Patients with an NLR < 5 both pre‐ and post‐nivolumab had significantly improved OS compared to patients with an NLR ≥ 5 pre‐ and posttreatment (Figure 2). There was also a significant inverse relationship between OS and PLR tertiles. PFS was significantly longer among patients with an NLR < 5 vs ≥ 5 pretreatment (16 vs 5 months, *P* = .022) as well as posttreatment (35 vs 5 months, *P* < .001). No significant differences in PFS among PLR tertiles pre‐ or posttreatment were observed. Posttreatment NLR and PLR appeared to be more strongly predictive of differences in survival than pretreatment values.

PLR and NLR were first added as continuous covariates to multivariable Cox proportional hazard models separately to assess the association between these two markers and OS. Pretreatment NLR did not show a significant association with survival (HR = 1.01 (95% CI: 1.00, 1.03); however, pretreatment PLR (HR = 1.001 (95% CI: 1.000, 1.002) was significantly associated with greater survival. Posttreatment PLR (HR = 1.002 (95% CI: 1.001, 1.004), *P* < .001) and NLR (HR = 1.10 (95% CI: 1.05, 1.15), *P* < .001) were significantly associated with OS in models that controlled for both Child‐Pugh class and BCLC stage in the models.

### 3.5 Statistical models by NLR/PLR groups

In the multivariable Cox PH model after controlling for baseline AFP, Child‐Pugh class, and BCLC stage, the high‐NLR/low‐PLR group was associated with a >2‐fold increased risk of death than the low‐NLR/low‐PLR group (HR = 2.18, (95% CI 1.16, 4.09) *P* = .016). The high‐PLR group was associated with an over eightfold greater risk of death than the low‐NLR/low‐PLR group (HR = 8.3 (95% CI 3.00, 22.99) *P* < .001) (Table 3).

**Table 3:** Multivariable model results for OS

	HR	95% CI	*P*‐value
Log10 Baseline AFP	1.52	(1.18, 1.95)	.001
BCLC			
C vs B	4.52	(1.56, 13.16)	.006
CPS			
B vs A	1.95	(1.06, 3.58)	.032
Post treatment NLR/PLR			
High NLR Low PLR vs Low NLR Low PLR	2.18	(1.16, 4.09)	.016
High PLR vs Low NLR Low PLR	8.30	(3.00, 22.99)	<.001

Abbreviations: BCLC, Barcelona Clinical Liver Cancer Staging; CPS, Child‐Pugh Stage.

## 4 DISCUSSION

### 4.1 Predictive immunotherapy biomarkers in HCC—an unmet need

Several Immunotherapy trials have demonstrated substantially higher response rates, greater tolerability, and a trend toward improved survival in favor of anti–PD‐1 therapy over sorafenib in HCC, driven by a subset of patients with clinically meaningful and durable responses. Analyses of the genomic landscape of HCC including TMB, PD‐1, DDR alterations have so far been unable to establish predictive biomarkers of response to anti–PD‐1 therapy.^7^, ^25^, ^26^, ^27^ Historically, diagnosis of HCC based on characteristic radiological findings on dynamic contract imaging has been repeatedly validated and is highly sensitive and specific, taking away the need for tissue diagnosis.^28^ Furthermore, imaging eliminates the morbidity and mortality associated with the biopsy procedure as well as the concern for tumor seeding; this however, also means there is often a lack of tumor tissue for molecular and microscopic biomarker analysis. It is thus imperative to explore nontissue based biomarkers, such as serum biomarkers which are readily available, cost effective, and easy to interpret.

This study to our knowledge is the first to evaluate the role of NLR, PLR, and their combination as predictive biomarkers of survival at baseline as well as after three cycles of anti–PD‐1 therapy in aHCC. It addresses the need for noninvasive biomarkers capable of stratifying aHCC patients based on their likelihood of responding to immunotherapy and for indicators of on‐treatment response.

### 4.2 Mechanistic rationale

**Figure 3:**
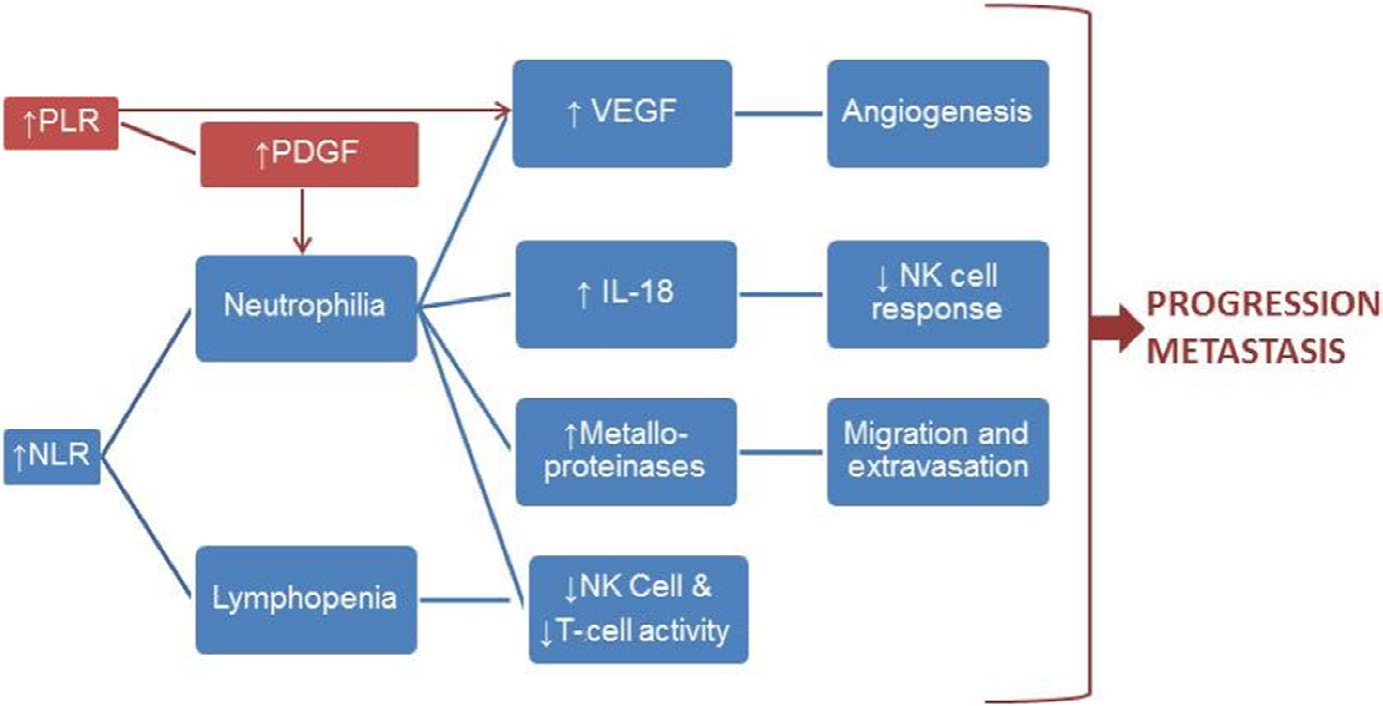
Rationale for predictive and prognostic role of NLR and PLR

The mechanistic rationale for the prognostic and predictive value of inflammatory markers in solid tumors has been well discussed in literature.^14^, ^29^, ^30^ Neutrophilia, as reflected in an elevated NLR, leads to increased production of neutrophil‐derived cytokines such as vascular endothelial growth factor (VEGF), matrix metalloproteinases, and interleukin‐18 (IL‐18). VEGF promotes angiogenesis and metalloproteinases increase extravasation and inflammation. IL‐18 impairs NK and T cell function, thus impairing host immune responses to tumor antigens.^31^ Thrombocytosis plays a similar role, mediated by VEGF and platelet‐derived growth factor (PDGF) production, which further recruits neutrophils and monocytes.^32^ These cascades of events thus promote tumor progression and metastasis (Figure 3).

## 5 RESULTS

In our analysis of 103 patients baseline AFP, CPS, and BCLC stage were all independently associated with survival. We demonstrate that an elevated NLR and PLR after completion of three cycles (6‐8 weeks) of nivolumab is strongly associated with worse clinical outcomes. Baseline NLR was not significantly associated with survival in our patient cohort, suggesting that a decrease in NLR after three cycles of nivolumab may be an early signal of clinic benefit from anti–PD‐1 therapy. In the posttreatment setting, lower NLR and PLR were not only independently predictive of improved survival, but also were associated with higher rates of partial and complete responses in patients with preserved liver function (Child‐Pugh A disease; Figure 1). The lack of a statistically significant association in Child‐Pugh B disease is likely attributable to an inadequately powered subset (n = 32). In a multivariable analysis of the study cohort, posttreatment NLR and PLR continued to demonstrate strong association with survival after controlling for major prognostic factors such as baseline AFP, Child‐Pugh score, and BCLC stage.

In an exploratory model evaluating the relationship between combined NLR and PLR and OS, there was an increased risk of death in patients with both elevated posttreatment NLR and PLR compared to those with either elevated NLR or PLR alone. Whether a composite NLR/PLR biomarker provides enhanced capacity to predict outcomes in HCC patients on immunotherapy will need to be further evaluated in a larger cohort of patients.

### 5.1 Thrombocytopenia as a confounder

Platelet count as a reflection of systemic inflammation in patients with cirrhosis is confounded by varying degrees of underlying portal hypertension. Therefore, a lower PLR may reflect both worsening cirrhosis, as well as decreased systemic inflammation. A threshold of PLR at which a lower platelet count may be detrimental to outcomes, driven by poor liver function could not be established in our Child‐Pugh A patient cohort, though a difference in posttreatment PLR was noted between response groups (Figure 1). This was due to an inadequate sample size and remains a subject of investigation be explored in a larger cohort.

### 5.2 Clinical Utility of NLR and PLR

In clinical practice, most patients undergo imaging after 4‐6 cycles of nivolumab (~2‐3 months from initiation of therapy) to evaluate for response to treatment. In patients with early signs of treatment failure such as worsening performance status, continued weight loss and cachexia, an elevated NLR and PLR at 6 weeks posttreatment may warrant early evaluation for progression of disease and consideration for early change in therapy.

### 5.3 Limitations

Limitations of our study include a relatively small sample size, other variables that were not evaluated, and its single‐institutional, nonprospective design. Our sample size precludes definitive conclusions especially in our statistical models by NLR/PLR groups, but can be considered hypothesis‐generating and warrants further validation in a large, prospective cohort. Data were collected and entered manually, but no missing data were noted on our analysis. A reviewer suggested other systemic inflammatory conditions such as Inflammatory Bowel Disease may affect NLR and PLR. However, apart from underlying Cirrhosis, history of other systemic inflammatory conditions was not collected during chart review and therefore cannot be evaluated.

## 6 CONCLUSIONS

In conclusion, these inflammatory cell ratios are promising biomarkers of outcome in aHCC patients receiving anti–PD‐1 therapy. In the posttreatment setting these ratios show strong association with survival and are readily available, economic and noninvasive tests that may be used as early predictor of treatment failure once validated in a larger, prospective cohort.

## AUTHORS CONTRIBUTION

**Sirish Dharmapuri**: Study concept and design, acquisition of data, analysis and interpretation of data, drafting of the manuscript, and critical revision of the manuscript for important intellectual content. **Umut Özbek**: Statistical analysis and critical revision of the manuscript for important intellectual content. **Jung‐Yi Lin**: Statistical analysis and critical revision of the manuscript for important intellectual content. **Max Sung**: Critical revision of the manuscript for important intellectual content and study supervision. **Myron Schwartz**: Critical revision of the manuscript for important intellectual content and study supervision. **Andrea D. Branch**: Study concept and design, and critical revision of the manuscript for important intellectual content. **Celina Ang**: Study concept and design, analysis and interpretation of data, drafting of the manuscript, and critical revision of the manuscript for important intellectual content, study supervision.

## Data Availability

The data that support the findings of this study are available from the corresponding author upon reasonable request.
